# Management of intraoperative bronchospasm

**DOI:** 10.4103/0019-5049.76587

**Published:** 2011

**Authors:** Indira Malik

**Affiliations:** Department of Anaesthesiolgy and Critical Care, Govind Ballabh Pant Hospital, New Delhi, India

Sir,

I read with interest the correspondence by Shukla.[1] titled “Intraoperative bronchospasm with thiopental” There are some essential concerns regarding the management of the case

First, the patient who presented in the emergency department for incision and drainage of a breast abscess was kept nil per orally for 6 hours and aspiration prophylaxis was also given. Since she gave a history of childhood asthma, the author could have planned for general anaesthesia using a supraglottic device like proseal laryngeal mask airway (LMA). This would have minimized the manipulation of a suspected hyperreactive airway.[2]

Second, when bronchospasm had developed following administration of thiopentone, the patient’s trachea was intubated after which inj. Hydrocortisone, inj. Dexamethasone and inj. Aminophylline were given. Then, maintenance of anaesthesia was done using O_2_, N_2_O and halothane. The first and foremost step in a case of intraoperative bronchospasm is to deepen the plane of anaesthesia.[3] So, they could have started halothane at a higher concentration than the one used since halothane itself is a potent bronchodilator.

Third, the management of bronchospasm could have been done with salbutamol puffs along with hydrocortisone and dexamethasone, instead of aminophylline. The literature suggests that if salbutamol puffs are unable to treat the bronchospasm, terbutaline or even deriphylline we can use after dilution in normal saline.[4] Aminophylline has fallen out of favour with most physicians due to its adverse effects. It is now used only as a last resort when no other drug is able to reverse bronchospasm.

Fourth, after giving the loading dose, infusion of aminophylline was started and, simultaneously, halothane was also being given to maintain anaesthesia. Aminophylline is a potential arrhythmogenic agent causing release of adrenaline and halothane sensitizes the myocardium to catecholamines.[5] So, the concurrent administration of these two agents is questionable
